# Allelopathic Effect of the Invasive Species *Acacia dealbata* Link and *Hakea decurrens* R.Br., subsp. *physocarpa* on Native Mediterranean Scrub Species

**DOI:** 10.3390/plants14233685

**Published:** 2025-12-03

**Authors:** Laura Nogales, Natividad Chaves, José Blanco-Salas, Laura Mateos, Luz Victoria Rubio, Juan Carlos Alías

**Affiliations:** Department of Plant Biology, Ecology and Earth Sciences, Faculty of Science, Universidad de Extremadura, 06001 Badajoz, Spain; lnogalesg@unex.es (L.N.); natchalo@unex.es (N.C.); blanco_salas@unex.es (J.B.-S.); lauramg@unex.es (L.M.); lurubioh@unex.es (L.V.R.)

**Keywords:** allelopathy, Mediterranean ecosystems, invasive species, scrub

## Abstract

Invasive species can profoundly alter ecosystems through mechanisms such as allelopathy. This study evaluates the allelopathic effects of *Acacia dealbata* and *Hakea decurrens* subsp. *physocarpa* on two dominant Mediterranean native species, *Cistus ladanifer* and *Lavandula stoechas*. Germination bioassays using aqueous extracts (1:10 *w*/*v*) at concentrations of 1, 1/2, and 1/4 of leaves collected in March and September were used to evaluate germination, hypocotyl emergence, and root development compared to control values (water) and between treatments. The phenolic composition of the solutions used was also analyzed. Significant inhibitory effects were observed across all parameters, especially at high concentrations, with responses modulated by the invasive species, the native target, and seasonal variation. *A. dealbata* showed stronger phytotoxicity in March, while *H. decurrens* subsp. *physocarpa* was more active in September. Phytochemical analysis revealed a higher load of phenolic compounds in *A. dealbata*, which may be related to the greater allelopathic activity of this species. These findings confirm the allelopathic potential of both invasive species and their ability to interfere with the establishment of native plants while facilitating their own, potentially impacting the colonization success of invasive species and altering vegetation succession in Mediterranean ecosystems.

## 1. Introduction

Biological invasions constitute one of the main threats to biodiversity conservation [1]. By altering community structure and ecosystem functioning, they reduce the abundance of native species [2]. They can even become exclusively established in the habitats they invade, completely displacing local species [3,4]. Among the mechanisms that may favor the success of certain invaders, allelopathy has gained relevance due to its potential to reconfigure communities and alter successional trajectories [5]. Allelopathy consists of the release of secondary metabolites that modify the germination, growth, or physiology of neighboring plants and/or their soil microbiomes [6,7,8]. It is very likely that allelopathic compounds produce both stimulating and inhibitory effects on native plants, but the direction of the interaction will depend on the compound concentration and the type and phenological stage of the native plants [9]. In turn, the Novel Weapons Hypothesis proposes that some invasive plants succeed in their colonizing power because they introduce chemical compounds to which native species lack tolerance due to the absence of a coevolutionary history [8,10]. All this supports the relevance of studying allelopathy in the context of biological invasions. Recent meta-analyses have revealed that a significant proportion of invasive plants exhibit allelopathic effects [11], although the actual prevalence may be underestimated due to the lack of specific studies [8].

In this context, *Acacia dealbata* Link (Fabaceae) and *Hakea decurrens* R.Br., subsp. *physocarpa* have emerged as high-impact invasive species in southwestern Europe, where they have colonized Mediterranean habitats and displaced native flora. In the Iberian Peninsula and other areas of the western Mediterranean, *A. dealbata* has rapidly expanded since its introduction as an ornamental species in the 19th century, forming monospecific stands. Belonging to the Fabaceae family, it is a tree native to southeastern Australia and Tasmania. It prefers temperate and humid climates at altitudes between 300 and 1000 m, tolerating poor soils [12]. It has invaded native forests, abandoned agricultural lands, and riparian zones [13], displacing native species [14]. Experimental evidence supports its allelopathic potential through the release of bioactive compounds [13,15,16]. Aqueous extracts from different plant parts of *A. dealbata* inhibit germination and growth of grassland species [17] and also reduce germination and root elongation in *Lactuca sativa* L. [13,18]. Phenolic compounds are likely responsible for these inhibitory effects, as complex phenolics have been reported in several *Acacia* species [18,19].

Similarly, *H. decurrens* subsp. *physocarpa*, another Australian shrubby species (0.8–4 m) with needle-like leaves of the Protaceae family, which inhabits temperate areas with warm, slightly humid summers and is well-adapted to drought and poor soils [20], has recently been identified as the predominant invader of Portugal and other European and African regions [21], replacing *Hakea sericea*, which had previously been more widely recognized and studied [22]. Its expansion toward southern areas of the Iberian Peninsula is well documented [23]. This species is associated with negative impacts on native vegetation and increased fire risk due to its pyrophytic traits and fruit serotiny [24]. Recent studies have demonstrated allelopathic activity in its leaves through aqueous extracts, which inhibit germination and growth of *L. sativa* L. [23], indicating potential allelopathic interference. However, no evidence exists regarding its effects on germination and development of native Mediterranean species within its invaded range in the Iberian Peninsula. Furthermore, several compounds (e.g., arbutin, phenolic derivatives, and glycosylated flavonoids) potentially involved in phytotoxicity have been identified. This set of findings positions *H. decurrens* subsp. *physocarpa* as a strong candidate for exerting allelopathic interference on Mediterranean shrublands.

Therefore, the aim of this study is to evaluate the allelopathic effect of *A. dealbata* and *H. decurrens* subsp. *physocarpa* on native Mediterranean species, integrating responses related to germination, early establishment, and growth, while considering seasonal variations in both chemical composition and phytotoxic effects. The two target native species selected for this study, *Cistus ladanifer* L. and *Lavandula stoechas* L., are structural components of thermo-Mediterranean shrublands, widely dominant in acidic soils and xeric environments of the Iberian Peninsula [25]. This approach will not only determine the potential impact of allelochemicals on the development of native species but also provide evidence of the ecological relevance of allelopathy as an invasion mechanism in Mediterranean ecosystems.

## 2. Results

### 2.1. Allelopathic Effect of Aqueous Extracts from Invasive Species on Native Mediterranean Species

#### 2.1.1. Effect on Germination and Hypocotyl Emergence

**Figure 1:**
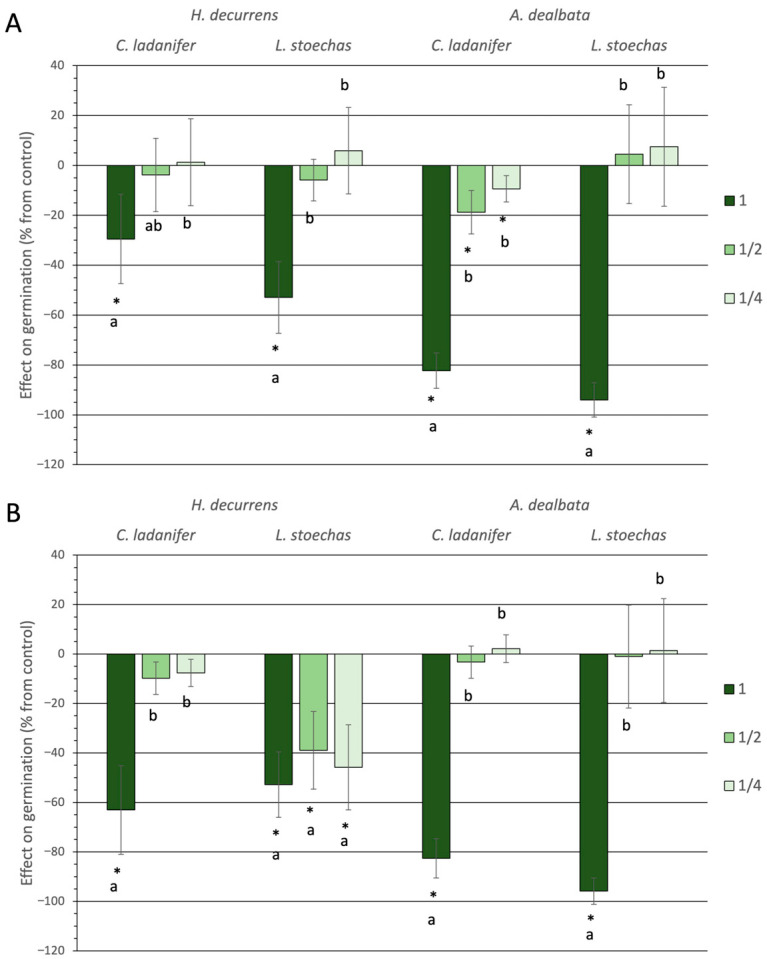
Effect on seed germination of *Cistus ladanifer* and *Lavandula stoechas* expressed as a percentage relative to the control, using aqueous extracts at different concentrations from leaves of *Hakea decurrens* subsp. *physocarpa* and *Acacia. dealbata* obtained in March (**A**) and September (**B**). *: significant differences compared to the control. a, b: different letters indicate significant differences (Tukey test, *p* < 0.05) between concentrations within the same treatment.

All tested aqueous solutions at high concentration caused a significant inhibition of germination (Tukey test, *p* < 0.05), with the effect of *A. dealbata* being particularly strong on both *C. ladanifer* and *L. stoechas*, reaching inhibition levels above 80% in both months (Figure 1). As concentration decreased, inhibition was generally lost, except for *A. dealbata* solutions in March on *C. ladanifer* seeds and *H. decurrens* subsp. *physocarpa* solutions in September on *L. stoechas* seeds. In these cases, even at the lowest concentration, significant inhibition was observed.

The statistical analysis of the inhibitory effect of the studied invasive species on germination percentages revealed significant differences between the two species (ANOVA, *p* = 0.048) (Table S1), with *A. dealbata* showing greater inhibition (Table S2). These differences were significant only in March (Tukey test, *p* < 0.001) and not in September (Tukey test, *p* = 0.365) (Table S3).

Regarding the sensitivity of native species, no significant differences were found in their germination response to the invasive species (ANOVA, *p* = 0.083) (Table S1). However, when analyzed separately, *C. ladanifer* exhibited significantly greater sensitivity to *A. dealbata* compared to *H. decurrens* subsp. *physocarpa* (Tukey test, *p* = 0.007) (Table S3). In contrast, *L. stoechas*, although apparently more sensitive to *A. dealbata*, did not show a significant difference (Tukey test, *p* = 0.94) (Table S3).

**Figure 2:**
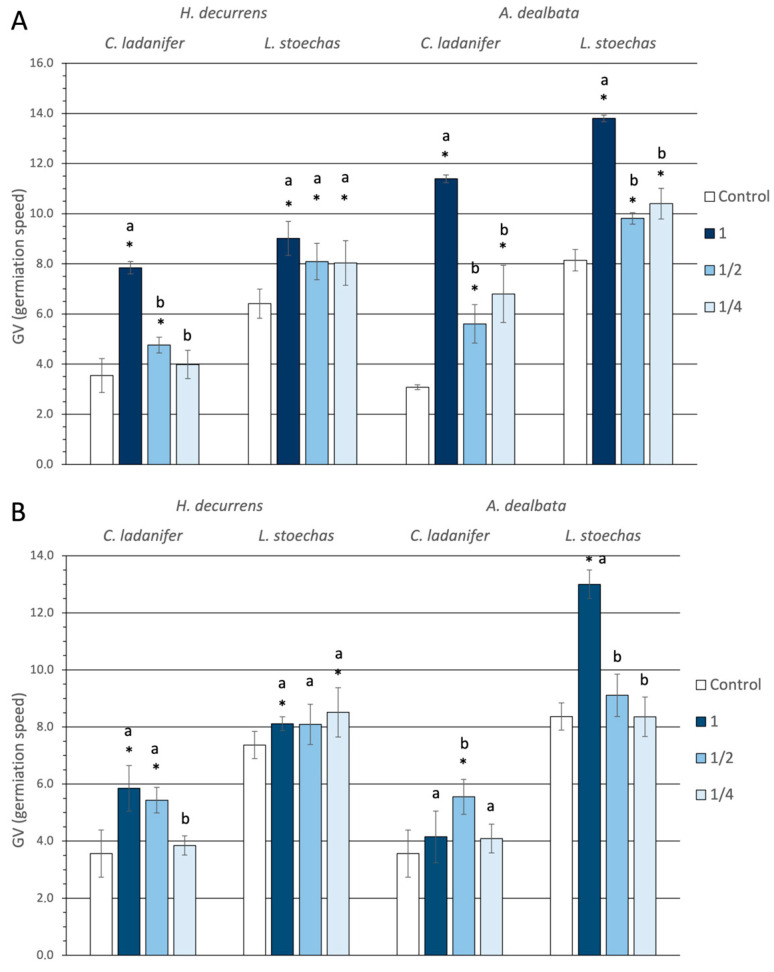
Germination rate expressed as the average number of days from the start of the experiment for seeds of *Cistus ladanifer* and *Lavandula stoechas* irrigated with aqueous extracts at different concentrations from leaves of *Hakea decurrens* subsp. *physocarpa* and *Acacia dealbata* collected in March (**A**) and September (**B**). * indicates significant differences compared to the control. Different letters (a, b) denote significant differences among concentrations within the same treatment (Tukey test, *p* < 0.05).

When analyzing the mean time required for seeds to germinate (Figure 2), a delay compared to the control was observed, particularly at high concentrations, but also at low concentrations in cases such as *H. decurrens* subsp. *physocarpa* solutions on *L. stoechas* in March and September, or *A. dealbata* solutions on *C. ladanifer* and *L. stoechas* in March. The greatest percentage delay relative to the control was caused by *A. dealbata* solution at high concentration on *C. ladanifer* and *L. stoechas* in March, with 280% and 70%, respectively. Additionally, *H. decurrens* subsp. *physocarpa* solution in March reduced the germination rate of *C. ladanifer* seeds by 112%.

When analyzing the effects on germination speed across different solutions, significant differences were found between the two invasive species (ANOVA, *p* < 0.001) (Table S4), with *A. dealbata* solutions causing greater delays than those of *H. decurrens* subsp. *physocarpa* (Table S5). This difference was significant only in March (Tukey test, *p* < 0.001) and not in September (Tukey test, *p* = 0.987) (Table S6). Moreover, the effect depended on the target species. Significant differences were observed between the effect of the two invasive species on germination delay of *C. ladanifer* seeds (Tukey test, *p* < 0.001), but not on *L. stoechas* seeds (Tukey test, *p* = 0.253) (Table S7).

Sensitivity in germination speed also differed between native species (ANOVA, *p* < 0.001) (Table S4), with *C. ladanifer* showing greater delays than *L. stoechas*, and these differences occurred in both tested months (Tukey test, *p* < 0.001) (Table S8).

**Figure 3:**
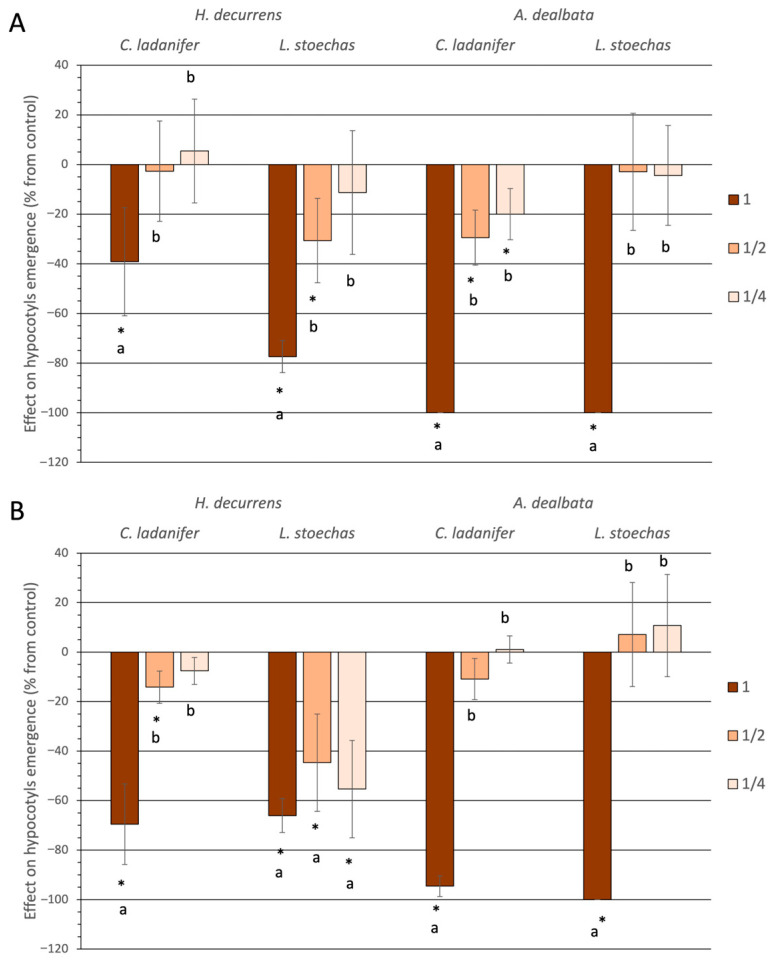
Effect on hypocotyl emergence in seeds of *Cistus ladanifer* and *Lavandula stoechas*, expressed as a percentage relative to the control, using aqueous extracts at different concentrations from leaves of *Hakea decurrens* subsp. *physocarpa* and *Acacia dealbata* collected in March (**A**) and September (**B**). * indicates significant differences compared to the control. Different letters (a, b) denote significant differences among concentrations within the same treatment (Tukey test, *p* < 0.05).

Hypocotyl emergence followed a similar pattern to germination, with concentration-dependent effects and significant inhibition mainly at high concentrations (Figure 3). The statistical analysis of the inhibitory effect of the invasive species on hypocotyl emergence percentages revealed no overall significant differences between the two species (ANOVA, *p* = 0.422) (Table S9), although differences were detected depending on the month: *A. dealbata* exerted a stronger inhibitory effect in March (Tukey test, *p* = 0.002), whereas *H. decurrens* subsp. *physocarpa* did so in September (Tukey test, *p* = 0.044) (Table S10).

Regarding the sensitivity of native species, significant differences were observed in their response to the invasive species (ANOVA, *p* = 0.015) (Table S9). Both *C. ladanifer* and *L. stoechas* showed greater reduction in hypocotyl emergence when treated with *A. dealbata* solutions compared to *H. decurrens* subsp. *physocarpa* solutions (Table S11).

**Figure 4:**
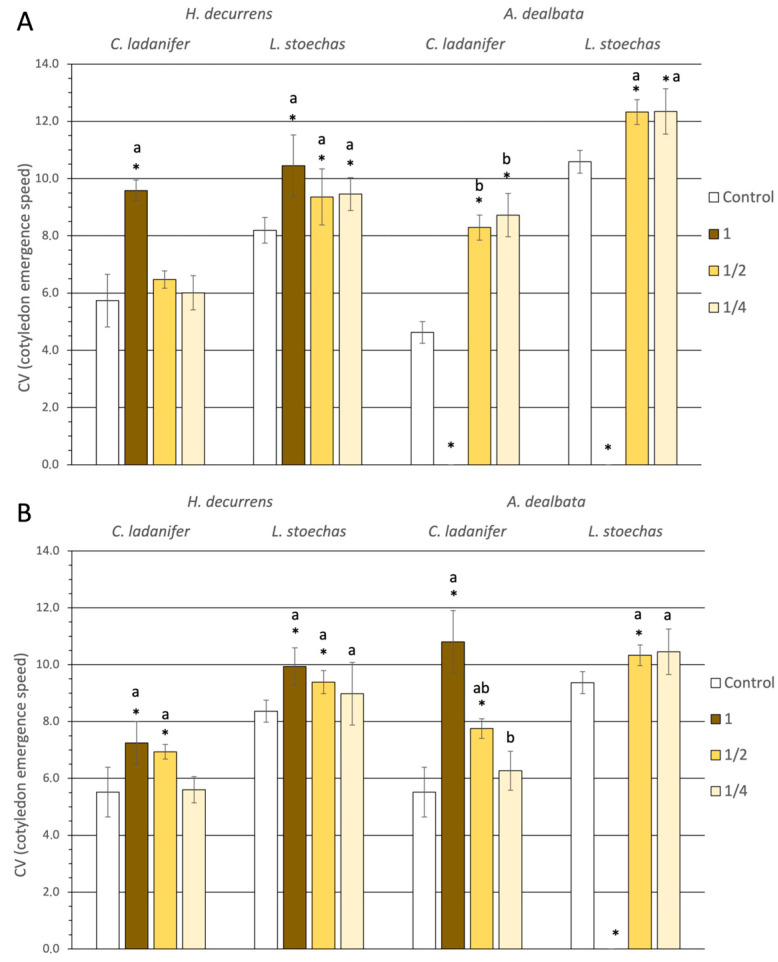
Hypocotyl emergence rate expressed as the average number of days from the start of the experiment for seeds of *Cistus ladanifer* and *Lavandula stoechas* irrigated with aqueous extracts at different concentrations from leaves of *Hakea decurrens* subsp. *physocarpa* and *Acacia dealbata* collected in March (**A**) and September (**B**). * indicates significant differences compared to the control. Different letters (a, b) denote significant differences among concentrations within the same treatment (Tukey test, *p* < 0.05).

When analyzing the mean time for hypocotyl emergence (Figure 4), a significant delay compared to the control was observed, particularly at high concentrations, but also at low concentrations in cases such as *H. decurrens* subsp. *physocarpa* solutions on *L. stoechas* in March and September, or *A. dealbata* solutions on *C. ladanifer* and *L. stoechas* in March. It is noteworthy that at concentration 1 of *A. dealbata*, germination was completely inhibited, so cotyledon emergence did not occur. Nevertheless, the greatest percentage delay relative to the control was caused by *A. dealbata* solution at concentration 1 on *C. ladanifer* in September, with 96%. Additionally, *H. decurrens* subsp. *physocarpa* solution at concentration 1 in March reduced cotyledon emergence speed in *C. ladanifer* by 69%.

Significant differences were observed between the two invasive species (ANOVA, *p* < 0.001) (Table S12), with *A. dealbata* solutions causing greater delays than those of *H. decurrens* subsp. *physocarpa* (Table S13) in both months analyzed (Table S14). Sensitivity in emergence speed did not differ between native species overall (ANOVA, *p* = 0.905) (Table S12), but when analyzed by month, the delay was significantly greater in *C. ladanifer* in March (Tukey test, *p* = 0.038), whereas in September the greatest delay occurred in *L. stoechas* (Tukey test, *p* = 0.024) (Table S15).

#### 2.1.2. Effect on Root Development

**Figure 5:**
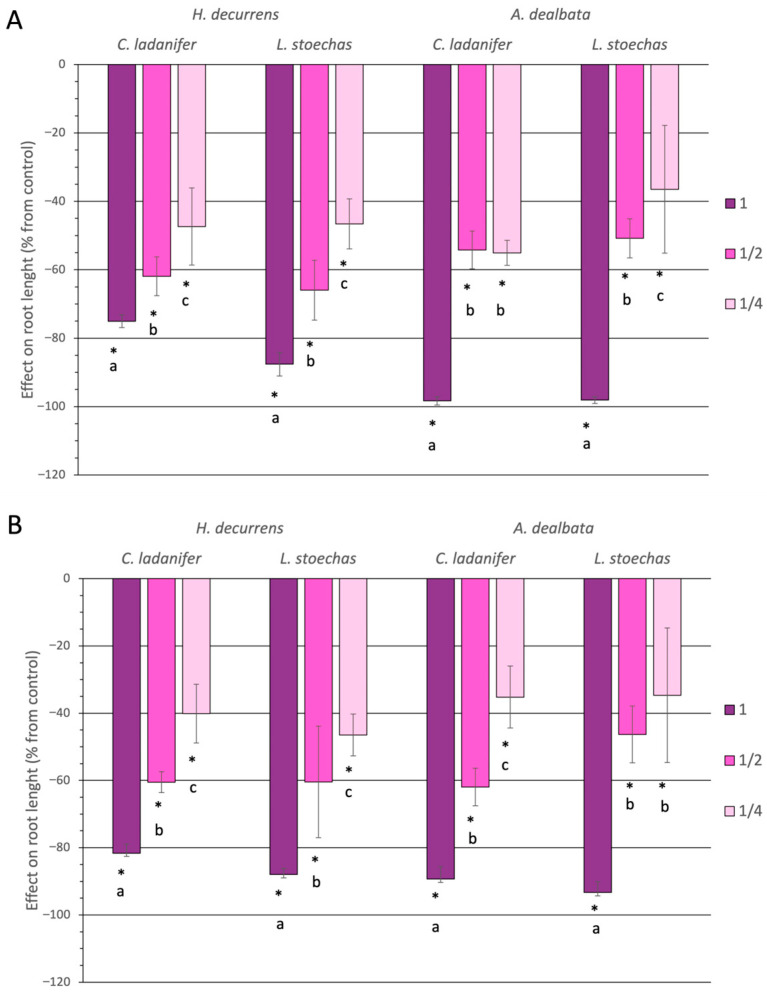
Effect on root length of *Cistus ladanifer* and *Lavandula stoechas* seedlings, expressed as a percentage relative to the control, using aqueous extracts at different concentrations from leaves of *Hakea decurrens* subsp. *physocarpa* and *Acacia dealbata* collected in March (**A**) and September (**B**). * indicates significant differences compared to the control. Different letters (a, b, c) denote significant differences among concentrations within the same treatment (Tukey test, *p* < 0.05).

Root length is a highly sensitive parameter to extract concentration, showing a progressive loss of inhibition as concentration decreases (Figure 5). Nevertheless, the inhibitory effect on root development was significant across all treatments, at all concentrations, and in both months (Tukey test, *p* < 0.05). At the highest concentration, root length was reduced by more than 80%, and even at one-quarter concentration, the reduction remained between 35% and 55%.

Both invasive species exhibited a similar overall effect on root length (ANOVA, *p* = 0.494) (Table S16), but the effect varied depending on the native species targeted. *H. decurrens* subsp. *physocarpa* caused greater inhibition on *L. stoechas* than on *C. ladanifer* (Tukey test, *p* < 0.001) (Table S17), although this difference was statistically significant only in September (Tukey test, *p* = 0.042) and not in March (Tukey test, *p* = 0.151) (Table S18). Conversely, *A. dealbata* inhibited root length of *C. ladanifer* more than that of *L. stoechas* (Tukey test, *p* = 0.004) (Table S17), regardless of the month (Table S18).

Finally, the overall sensitivity of native species was similar (ANOVA, *p* = 0.647) (Table S16); however, it depended on the invasive species and the month analyzed. *C. ladanifer* was more affected by *A. dealbata* (Tukey test, *p* = 0.007) (Table S17), but only in March (Tukey test, *p* = 0.011) (Table S18), whereas *L. stoechas* was more affected by *H. decurrens* subsp. *physocarpa* (Tukey test, *p* < 0.001) (Table S17), specifically in September (Tukey test, *p* = 0.003) (Table S18).

### 2.2. Phytochemical Analysis of Aqueous Extracts

**Figure 6:**
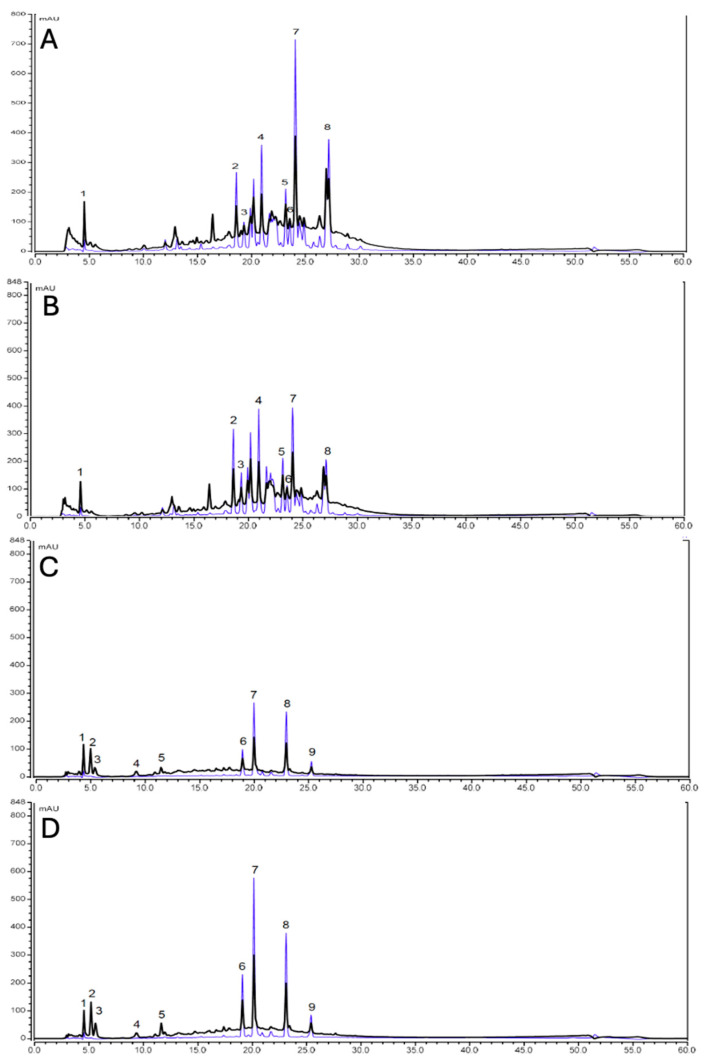
HPLC chromatogram of the aqueous extract of *Acacia dealbata* in March (**A**) and September (**B**); and of *Hakea decurrens* subsp. *physocarpa* in March (**C**) and September (**D**), at 280 nm (black line) and 350 nm (blue line). (**A**,**B**): 1: gallic acid; 2: Quercetin-O-dihexoside; 3: Quercetin-3,7-diglucoside; 4: Quercetin 3-rhamninoside; 5: Myricetin-3-arabinoside; 6: rutin; 7: isoquercetin; 8: quercitrin. (**C**,**D**): 1: arbutin; 2: mesaconic acid; 3: isotachioside; 4: 1-O-vanilloyl-beta-D-glucose; 5: syringic acid-4-beta-D-glucopyranoside; 6: quercetin 3-robinobioside-7-glucoside; 7: quercetin 3-rhamninoside; 8: rutin; 9: isorhamnetin-3-O-rutinoside.

Following chromatographic analysis of the aqueous extracts, the major compounds present in the different extracts were identified (Figure 6).

Chromatographic analysis of the aqueous extracts revealed eight predominant compounds in *A. dealbata*: gallic acid, quercetin-O-dihexoside, quercetin-3,7-diglucoside, quercetin-3-rhamninoside, myricetin-3-arabinoside, rutin, isoquercetin, and quercitrin. In contrast, nine major constituents were identified in *H. decurrens* subsp. *physocarpa*: arbutin, mesaconic acid, isotachioside, 1-O-vanilloyl-β-D-glucose, syringic acid-4-β-D-glucopyranoside, quercetin-3-robinobioside-7-glucoside, quercetin-3-rhamninoside, rutin, and isorhamnetin-3-O-rutinoside.

Although the number of identified compounds was similar between species, the total concentration of phytochemicals was approximately threefold higher in *A. dealbata* compared to *H. decurrens* subsp. *physocarpa*. Notably, rutin and quercetin-3-rhamninoside were common to both species. Quantitative analysis indicated that the overall phytochemical content varied depending on the collection month of the aqueous extracts. While *A. dealbata* exhibited no significant seasonal variation, *H. decurrens* subsp. *physocarpa* showed a higher concentration of compounds in December compared to March (Table 1).

## 3. Discussion

The results obtained in this study confirm that *A. dealbata* and *H. decurrens* subsp. *physocarpa* exert significant phytotoxic effects on native Mediterranean species such as *C. ladanifer* and *L. stoechas*. These effects manifest during several stages of early plant development, including germination, hypocotyl emergence, and root growth, potentially reducing competition with native species. Moreover, the response is modulated by extract concentration and is seasonally dependent.

All parameters analyzed in the target species were significantly affected at high extract concentration. In fact, this concentration (1:10 *w*/*v*) is among the most commonly used in allelopathic studies as a starting point for bioassays [18,26]. Nevertheless, it has also been demonstrated that even at lower concentrations (1:40 *w*/*v*), the extracts exert phytotoxic effects on germination, hypocotyl emergence, and particularly on root development. Therefore, the responses observed cannot be attributed exclusively to high dosages. Moreover, laboratory assays conducted with extracts may constitute a valid approximation for assessing the allelopathic potential of the studied species, since several studies have reported a strong correlation between results obtained under controlled conditions and those observed in the field [27,28]. However, this does not preclude the necessity of an ecological validation, in which concentrations should be adjusted to reflect actual levels of field incorporation. Indeed, assays using rainfall leachates of *A. dealbata* have demonstrated the allelopathic potential of these leachates [13], which highlights the importance of quantifying compounds mobilized by rainfall, both in *A. dealbata* and *H. decurrens* subsp. *physocarpa*, to determine the extent of their phytotoxicity under natural conditions. Furthermore, the high solubility of the quantified compounds in aqueous extracts (Table 1) confirms that these metabolites can be readily mobilized by precipitation, thus providing an experimental framework closely aligned with natural conditions. Finally, it should be noted that plant residues represent another important pathway for the incorporation of allelopathic compounds into the soil, releasing lipophilic fractions as well [8]. Consequently, this aspect should be considered in future studies to quantify the total allelopathic activity of these two invasive species.

In addition to the negative effects of aqueous extracts on germination, it is worth noting that alterations in germination rate and hypocotyl emergence are particularly relevant, as they may limit seedling establishment under climatically stressful environments such as the Mediterranean, where optimal establishment conditions are not always present. Indeed, studies conducted by Wang et al. (2022) [28] highlighted that seed germination rate may represent one of the most important allelopathic indices. Therefore, this parameter can effectively reflect germination performance when assessing allelopathic potential. Similarly, root length, as a measure of root system development, is crucial for seedling viability [29], since the root is the primary organ regulating water and nutrient absorption and conferring resistance to stress and adverse habitats. Nevertheless, the marked sensitivity of roots to allelopathy underscores their utility in allelopathy detection [30,31]. In our study, the inhibitory effect observed—approximately 40% reduction in root length at low concentrations—further reinforces the allelopathic potential of both invasive species examined.

Therefore, our results clearly highlight the phytotoxicity of *A. dealbata* on the two native species studied. Previous studies have documented the strong allelopathic potential of *A. dealbata* through assays conducted on *L. sativa* seeds [13,14,15,16]. Conversely, when field trials have been performed to evaluate the role of allelopathy in its expansion capacity, the effects have not been as evident, even leading to the exclusion of its involvement in colonization success [32]. To reach this conclusion, the authors tested native species such as *Trifolium angustifolium*, *Pinus pinaster*, and *Cytisus striatus* in a specific region of the Iberian Peninsula characterized by a Eurosiberian biogeographic origin and a humid Atlantic climate. In contrast, in our study both the target species and the *Acacia* plant material samples originate from the Mediterranean region with a thermo-Mediterranean climate. Consequently, their responses do not necessarily have to be compatible [18].

Although *H. decurrens* subsp. *physocarpa* has been less studied, the results presented here, together with recent findings by Nogales et al. (2025) [23], indicate that it also exhibits a relevant allelopathic profile, particularly in September, suggesting seasonal variability possibly linked to phytochemical composition. Indeed, this variability is reflected in the metabolite composition analyzed, where an increase in compounds was observed in September compared to March. Moreover, the seasonal variation observed in the effects of the extracts is consistent with studies on other species such as *Brachiaria brizantha*, *Pinus densiflora*, and *C. ladanifer*, where allelochemical concentrations vary significantly throughout the year [32,33,34,35]. This strongly suggests that the production of secondary metabolites is regulated by phenological and environmental factors. In addition, the variation in total compound content between March and September in *H. decurrens* subsp. *physocarpa* extracts appears to be related to the differences observed in the phytotoxic capacity of both species. Specifically, in March, *A. dealbata* extracts displayed significantly higher phytotoxic activity than those of *H. decurrens* subsp. *physocarpa*, coinciding with a marked difference in compound concentration (12.7 mg/gPS in *A. dealbata* versus 3.54 mg/gPS in *H. decurrens*). By September, although *A. dealbata* maintained higher values (12.46 mg/gPS versus 5.99 mg/gPS in *H. decurrens* subsp. *physocarpa*), the disparity was less pronounced. Consequently, the greater difference in compound concentration recorded in March may explain the superior phytotoxic capacity of *A. dealbata* during that period.

Native species such as *C. ladanifer* and *L. stoechas* also exhibit differential tolerance or sensitivity to the phytotoxicity of invasive species. Specifically, *C. ladanifer* showed greater sensitivity in terms of delayed germination and reduced root growth in response to *A. dealbata* in March, whereas *L. stoechas* was more affected by *H. decurrens* subsp. *physocarpa* in September. This behavior is consistent with previous studies, which indicate that allelopathic effects depend on the recipient species, its phenological stage [9,36], and even its biogeographic origin [37,38]. Moreover, each receptor species may respond differently to donor extracts [39]. In general, studies on the effects of plant extracts on seedling growth of co-occurring species typically report inhibitory effects, although the degree of susceptibility is species-specific [40]. Consequently, this specificity suggests that compounds released by each invasive species interact differently with the physiology of native species [36].

HPLC analysis of aqueous extracts from *A. dealbata* and *H. decurrens* subsp. *physocarpa* showed higher diversity and concentration of secondary metabolites in *A. dealbata*. Flavonoids, particularly quercetin derivatives, were predominant in both species. Flavonoids are known to inhibit germination and seedling growth and are considered major allelochemicals responsible for physiological suppression in recipient species [41,42]. They interfere with cell division, elongation, membrane permeability, photosynthesis, respiration, and enzymatic activity [43], likely through interactions with proteins involved in mitosis and cell wall biogenesis [44].

Gallic acid, rutin, and arbutin were among the metabolites identified with documented allelopathic activity. Gallic acid inhibits *Brassica juncea* growth and causes root phytotoxicity in *Phragmites australis* by generating reactive oxygen species (ROS) [45,46]. Rutin strongly inhibits shoot growth, with negative correlations between its concentration and root and shoot length; it has been reported as a potent inhibitor in lettuce seedlings [47]. Arbutin has been linked to allelopathic activity in *Arbutus unedo* and *Myrtus communis* [48] and may also contribute to the phytotoxicity of *H. decurrens* subsp. *physocarpa* on *L. sativa* germination and root growth [23].

Nevertheless, although the phytotoxicity of these compounds is well established, attributing the allelopathic activity of these invasive species exclusively to individual metabolites is reductionist. Evidence indicates that extract toxicity depends not only on the nature and concentration of each compound but also on their interactions, which may be synergistic or antagonistic, as well as on their bioavailability [49,50]. For example, positive correlations have been described between rutin, isoquercetin, and astragalin, suggesting possible synergistic interactions in the phytotoxicity of these flavonoids [47]. Consequently, the phytotoxicity observed should be interpreted as the outcome of a complex network of chemical interactions rather than the isolated effect of a single metabolite.

The observed effects on germination and early development have direct implications for plant succession dynamics and the structure of Mediterranean communities [51]. As with *A. dealbata*, this allelopathic interaction in early developmental stages can alter natural regeneration and the resilience of ecosystems to disturbances such as fires [52]. In this sense, pyrophytic characteristics such as the serotiny of *H. decurrens* subsp. *physocarpa* increase the risk of fire for these ecosystems upon their establishment [24].

The inhibition of dominant species such as *C. ladanifer* and *L. stoechas* may facilitate the formation of monospecific stands of invasive species [53,54], as occurs with other well-studied species such as *Artemisia vulgaris*, *Lantana camara*, and *Medicago arborea* [55,56,57]. Studies such as that by Cipollini and Greenawalt (2016) [58] have shown that species like *Lonicera maackii* and *Celastrus orbiculatus* can reduce germination and biomass of native species by up to 80%. Similarly, *Cynara cardunculus* can inhibit, through the release of allelochemicals into the soil, the germination and development of the non-native species *Juncus pallidus* compared to the native grass species *Lolium rigidum* [59]. This behavior suggests a coevolutionary adaptation mediated by chemical interactions [60].

Although the study provides robust evidence of allelopathic effects and the differences observed between both species, it would be advisable to broaden the range of native species evaluated and conduct field trials to validate the ecological relevance of these results in a Mediterranean context.

## 4. Materials and Methods

### 4.1. Collection of Plant Material and Sample Preparation

Plant material was collected from invaded areas in Extremadura (Spain). *H. decurrens* subsp. *physocarpa* was specifically located in the municipality of Valverde del Fresno (40°13′26″ N, 6°52′47″ W), while *A. dealbata* was found in La Codosera (39°11′14″ N, 7°10′30″ W). The predominant vegetation associated with *H. decurrens* subsp. *physocarpa* consists of *Pinus pinaster*, with an understory mainly composed of *Cistus ladanifer*, *Lavandula stoechas*, *Arbutus unedo*, and *Calluna vulgaris* [61]. In contrast, *A. dealbata* forms dense stands, although surrounded by oak and cork oak formations with an understory primarily of *C. ladanifer* and *L. stoechas*. In 2024, leaf samples from both species were collected in March (late winter) and September (late summer), periods in which the leaves contain the synthesized compounds that interact with the native seeds during their germination phase (April-May for *L. stoechas* and October-November for *C. ladanifer*). Samples were obtained from different randomly selected adult individuals. Voucher specimens were deposited in the Herbarium of the Agricultural Research Institute Finca La Orden—Valdesequera, CICYTEX-Junta de Extremadura (HSS 87165/87181/87203), and subsequently pooled to obtain approximately 1 kg of leaves. In the laboratory, leaves were mixed and air-dried at room temperature, ground into powder using an electric grinder, and stored in darkness until further use. Seeds of the target species, *C. ladanifer* and *L. stoechas*, were commercially sourced.

### 4.2. Preparation of Aqueous Extracts

The dried, ground leaves were mixed with distilled water (1:10 *w*/*v*) [62,63] and kept under agitation at room temperature for 24 h. The mixture was then filtered, and three concentrations were prepared. The original solution (100%) was diluted with distilled water to obtain concentrations of 50% and 25%.

### 4.3. Biological Assays

The allelopathic effect of *H. decurrens* subsp. *physocarpa* and *A. dealbata* on accompanying shrub species was quantified through germination tests using seeds of *C. ladanifer* and *L. stoechas*. *Seeds of C. ladanifer* were subjected to a pre-germination treatment consisting of a heat shock at 100 °C for 5 min. Seeds of *L. stoechas* received no pre-germination treatment. Twenty-five seeds were placed in Petri dishes lined with filter paper (four replicates per species and concentration). Five milliliters of each dilution was added to the dishes, which were sealed with Parafilm. Distilled water was used as the control. The dishes were randomly arranged in a growth chamber at 20/15 °C, with a photoperiod of 14 h light and 10 h darkness. Germination and hypocotyl emergence were recorded daily for each dish until no changes occurred in the controls. At the end of the experiment, root length was measured in 10 seedlings per dish. Based on these data, the following parameters were calculated [64]:

- Germination percentage relative to control (%G): Number of seeds germinated in a given treatment relative to the average number of seeds germinated in the control group:

$$\%G=\left(\frac{\sum_{i=1}^{n}\frac{Gi}{GCn}}{n}\right)\times 100,$$

where *G_i_* is the number of germinated seeds in dish *i* (*i* = 1 to *n*), *GC_n_* is the mean number of germinated seeds in the four control dishes, and *n* is the number of dishes.

- Hypocotyl emergence percentage relative to control (%C): Calculated using the same formula as germination, replacing germinated seeds with emerged hypocotyls.
- Germination velocity (GV): Arithmetic mean indicating the number of days required for germination, calculated as:

$$GV=\frac{\sum_{i=1}^{n}\left(Ni\times Gi\right)}{\sum_{i=1}^{n}Gi},$$

where *N_i_* represents the days elapsed since the start of the test, and *G_i_* the number of seeds germinated each day (*i* = 1 to *n*).

- Hypocotyl emergence velocity (CV): Calculated using the same formula as GV, replacing germination with hypocotyl emergence.
- Root length relative to control (%LR): Average root length expressed as a percentage of the control, calculated as:

$$\%LR=\left(\frac{\sum_{i=1}^{n}Li}{\sum_{i=1}^{n}LCi}\right)\times 100,$$

where *L_i_* is the root length of each plant measured under a given treatment, *LC_i_* is the root length of each plant measured under control treatment, and *n* is the number of plants measured.

### 4.4. Identification and Quantification of Phenolic Compounds

#### 4.4.1. Identification: UHPLC/Q-TOF MS Method

Aqueous solutions were analyzed using a UHPLC system (Agilent 1260, Agilent Technologies, Santa Clara, CA, USA) equipped with a DAD detector (Agilent G7117A) and a QTOF mass analyzer (Agilent 6520) with electrospray ionization at atmospheric pressure (ESI). Separation was performed following the method described by Nogales et al., 2025 [23], using a reverse-phase C18 Spherisorb column (150 × 4.6 mm) (Waters corporation, Tauton, MA, USA). The mobile phase consisted of 0.1% formic acid in water (A) and 0.1% formic acid in acetonitrile (B) at a flow rate of 0.5 mL/min, applying the following gradient: initially 95% A; 10 min, 85% A; 20 min, 80% A; 40 min, 60% A; 50 min, 20% A; 55 min, 10% A; 65 min, 95% A. Data were acquired within a mass range of 100–1700 *m*/*z*, with a source temperature of 300 °C and a gas flow of 10 L/h. Molecular formulas proposed by MassHunter Workstation software version 4.0 for the different MS signals were compared with previously reported phenolic compounds, accepting a maximum error of 10 ppm. Mass measurement error (mass accuracy) was calculated according to Brenton and Godfrey [65]. Difference between an individual measurement and the true value ΔMi (in ppm, parts per million) = (M measured − M calculated) × 106/M calculated, where M measured is the measured mass in QTOF-MS, and M calculated is the exact calculated mass according to the molecular formula of the compound.

#### 4.4.2. Quantification: HPLC-DAD Method

Quantification of each identified phenolic compound was performed using HPLC (Agilent 1260, Agilent Technologies, Santa Clara, CA, USA) with a DAD detector (Agilent G7117A). Three replicates of 20 µL of filtered extract from each sample were injected. The column and chromatographic conditions were the same as those used for the identification process described above (Nogales et al., 2025) [23]: a Spherisorb C18 (150 × 4.6 mm) (Waters corporation, Tauton, MA, USA) reversed phase column at a rate of 0.5 mL/min. The mobile phase consisted of 0.1% formic acid in water (A) and 0.1% formic acid in acetonitrile (B) using a gradient as follows: initially, 95% A; 10 min 85% A; 20 min 80% A; 40 min 60% A; 50 min 20% A; 55 min 10% A; 65 min 95% A. Chromatograms were recorded at 350 nm and 280 nm. Calibration curves (0.001, 0.005, 0.05, 0.1, and 1 mg/mL) using gallic acid and quercetin 3-O-rutinoside (rutin) were employed to quantify phenolic acids and flavonoids, respectively. Results are expressed as mg equivalents per g of dry weight.

### 4.5. Statistical Analysis

The Kolmogorov–Smirnov test was used to assess the distribution of the data, and Levene’s test was applied to verify the homogeneity of variances. Subsequently, a multifactorial ANOVA for independent measures (GLM) was performed to analyze the main factors and their interactions. When significant differences were detected, Tukey’s HSD test was applied for pairwise comparisons. All statistical analyses were conducted using SPSS software (version 29.0.1.0). A *p*-value < 0.05 was considered statistically significant. Some of the results from the statistical analyses are provided in tables in the Supplementary Materials.

## 5. Conclusions

The findings of this study provide strong evidence that *A. dealbata* and *H.a decurrens* subsp. *physocarpa* exert significant allelopathic effects on key Mediterranean native species such as *C. ladanifer* and *L. stoechas*. These effects occur at multiple stages of early plant development, germination, hypocotyl emergence, and root growth, and are modulated by extract concentration and seasonal variability. Even at low concentrations, both species inhibited germination rates and root elongation, with *A. dealbata* showing greater phytotoxic potential, likely due to its higher diversity and concentration of phenolics compounds.

The observed inhibition of dominant natives could facilitate the formation of monospecific stands of these invasive species, as documented for other aggressive invaders. Differential sensitivity between *C. ladanifer* and *L. stoechas* underscores the species-specific nature of allelopathic interactions.

Overall, these results highlight the ecological relevance of allelopathy in invasion dynamics and suggest that chemical mediation may play a key role in competitive displacement. Future research should expand the range of native species tested and incorporate field-based experiments to validate these findings under natural Mediterranean conditions.

## Figures and Tables

**Table 1:** Total quantity of major compounds present in aqueous extracts of *Acacia deabata* and *Hakea decurres* subsp. *physocarpa* isolated and quantified by HPLC from samples obtained in March and September.

Total Compounds (mg/g DW)	March	September
*H. decurrens*	3.54	5.99
*A. dealbata*	12.70	12.46

## Data Availability

Data are contained within the article and Supplementary Materials.

## Supplementary Materials

The following supporting information can be downloaded at: https://www.mdpi.com/article/10.3390/plants14233685/s1, Table S1: Results of the ANOVA analysis examining the effects of invasive species, native species, concentration, month, and their combined interaction on the germination of native species seeds; Table S2: Results of Tukey’s HSD test assessing differences in the allelopathic effects of invasive species on the germination; Table S3: Results of Tukey’s HSD test identifying differences in the allelopathic effects of invasive species on seed germination depending on the native species; Table S4: Results of the ANOVA analysis examining the effects of invasive species, native species, concentration, month, and their combined interaction on the germination rate of native species seeds; Table S5: Results of Tukey’s HSD test assessing differences in the allelopathic effects of invasive species on the germination rate of native species; Table S6: Results of Tukey’s HSD test identifying significant differences in the allelopathic effects of invasive species on seed germination rate depending on the month of origin of the plant material; Table S7: Results of Tukey’s HSD test identifying differences in the allelopathic effects of invasive species on seed germination rate depending on the native species; Table S8: Results of Tukey’s HSD test identifying significant differences in the germination ratio of native species depending on the month of origin of the plant material; Table S9: Results of the ANOVA analysis examining the effects of invasive species, native species, concentration, month, and their combined interaction on the hypocotyl emergence of native species seeds; Table S10: Results of Tukey’s HSD test identifying significant differences in the allelopathic effects of invasive species on hypocotyl emergence depending on the month of origin of the plant material; Table S11: Results of Tukey’s HSD test identifying differences in the allelopathic effects of invasive species on hypocotyl emergence depending on the native species; Table S12: Results of the ANOVA analysis examining the effects of invasive species, native species, concentration, month, and their combined interaction on the hypocotyl emergence rate of native species seeds; Table S13: Results of Tukey’s HSD test assessing differences in the allelopathic effects of invasive species on the hypocotyl emergence rate of native species; Table S14: Results of Tukey’s HSD test identifying significant differences in the allelopathic effects of invasive species on hypocotyl emergence rate depending on the month of origin of the plant material; Table S15: Results of Tukey’s HSD test identifying significant differences in the hypocotyl emergence rate of native species depending on the month of origin of the plant material; Table S16: Results of the ANOVA analysis examining the effects of invasive species, native species, concentration, month, and their combined interaction on the root length of native species seeds; Table S17: Results of Tukey’s HSD test identifying differences in the allelopathic effects of invasive species on root length depending on the native species; Table S18: Results of Tukey’s HSD test identifying differences in the allelopathic effects of invasive species on root length as a function of native species and the month of origin of the plant material.
