# Supplementary material for: Allelopathic Effect of the Invasive Species Acacia dealbata Link and Hakea decurrens R.Br., subsp. physocarpa on Native Mediterranean Scrub Species
Source: Plants (Basel). 2025 Dec 3;14(23):3685. doi: 10.3390/plants14233685 (PMC12693743; doi:10.3390/plants14233685)
Supplement: Supplementary file 1 [file plants-14-03685-s001.zip › plants-3995591-supplementary.pdf]

Supplementary material

Table S1: Results of the ANOVA analysis examining the effects of invasive species, native species, concentration, month, and their combined interaction on the germination of native species seeds

ANOVA - Germination

	Sum of Squares	df	Mean Square	F	p
**Invasive species**	764.22	1	764.22	3.87533	0.048
**Native species**	607.62	1	607.62	3.08124	0.083
**Concentration**	81172.62	2	40586.31	205.81140	<.001
**Month**	2342.94	1	2342.94	11.88096	<.001
**Invasive species ✻ Native species**	1521.00	1	1521.00	7.71291	0.007
**Invasive species ✻ Concentration**	13467.10	2	6733.55	34.14555	<.001
**Native species ✻ Concentration**	285.33	2	142.66	0.72345	0.489
**Invasive species ✻ Month**	3616.95	1	3616.95	18.34140	<.001
**Native species ✻ Month**	943.51	1	943.51	4.78450	0.032
**Concentration ✻ Month**	196.89	2	98.45	0.49922	0.609
**Invasive species ✻ Native species ✻ Concentration**	1442.76	2	721.38	3.65809	0.031
**Invasive species ✻ Native species ✻ Month**	1.40	1	1.40	0.00711	0.933
**Invasive species ✻ Concentration ✻ Month**	303.44	2	151.72	0.76935	0.467
**Native species ✻ Concentration ✻ Month**	2508.71	2	1254.36	6.36079	0.003
**Invasive species ✻ Native species ✻ Concentration ✻ Month**	961.31	2	480.66	2.43738	0.095
**Residuals**	14198.51	72	197.20		

Table S2: Results of Tukey's HSD test assessing differences in the allelopathic effects of invasive species on the germination

Post Hoc Comparisons - Invasive species

Comparison							
Invasive species		Invasive species	Mean Difference	SE	df	t	p_{tukey}
**H. decurrens**	-	**A. dealbata**	5.64	2.87	72.0	1.97	0.048

Table S3: Results of Tukey's HSD test identifying differences in the allelopathic effects of invasive species on seed germination depending on the native species

Post Hoc Comparisons - Invasive species ✻ Native species

Comparison									
Invasive species	Native species		Invasive species	Native species	Mean Difference	SE	df	t	p_{tukey}
**H. decurrens**	**C. ladanifer**	-	**H. decurrens**	**L. stoechas**	12.992	4.05	72.0	3.205	0.011
		-	**A. dealbata**	**C. ladanifer**	13.604	4.05	72.0	3.356	0.007
		-	**A. dealbata**	**L. stoechas**	10.675	4.05	72.0	2.633	0.050
	**L. stoechas**	-	**A. dealbata**	**C. ladanifer**	0.611	4.05	72.0	0.151	0.999
		-	**A. dealbata**	**L. stoechas**	-2.318	4.05	72.0	-0.572	0.940
**A. dealbata**	**C. ladanifer**	-	**A. dealbata**	**L. stoechas**	-2.929	4.05	72.0	-0.723	0.888

Table S4: Results of the ANOVA analysis examining the effects of invasive species, native species, concentration, month, and their combined interaction on the germination rate of native species seeds

ANOVA - GV

	Sum of Squares	df	Mean Square	F	p
**Invasive species**	15897	1	15897	67.41	<.001
**Native species**	45629	1	45629	193.48	<.001
**Concentration**	44517	2	22259	94.38	<.001
**Month**	44920	1	44920	190.47	<.001
**Invasive species ✻ Native species**	7069	1	7069	29.97	<.001
**Invasive species ✻ Concentration**	3774	2	1887	8.00	<.001
**Native species ✻ Concentration**	9943	2	4971	21.08	<.001
**Invasive species ✻ Month**	17785	1	17785	75.41	<.001
**Native species ✻ Month**	15857	1	15857	67.24	<.001
**Concentration ✻ Month**	22897	2	11449	48.55	<.001
**Invasive species ✻ Native species ✻ Concentration**	2466	2	1233	5.23	0.008
**Invasive species ✻ Native species ✻ Month**	18535	1	18535	78.59	<.001
**Invasive species ✻ Concentration ✻ Month**	4458	2	2229	9.45	<.001
**Native species ✻ Concentration ✻ Month**	18689	2	9344	39.62	<.001
**Invasive species ✻ Native species ✻ Concentration ✻ Month**	6478	2	3239	13.73	<.001
**Residuals**	16037	68	236		

Table S5: Results of Tukey's HSD test assessing differences in the allelopathic effects of invasive species on the germination rate of native species

Post Hoc Comparisons - Invasive species

Comparison							
**Invasive species**		**Invasive species**	**Mean Difference**	**SE**	**df**	**t**	p_{tukey}
**H. decurrens**	-	**A. dealbata**	-26.8	3.26	68.0	-8.21	<.001

Table S6: Results of Tukey's HSD test identifying significant differences in the allelopathic effects of invasive species on seed germination rate depending on the month of origin of the plant material

Post Hoc Comparisons - Invasive species ✻ Month

Comparison									
**Invasive species**	**Month**		**Invasive species**	**Month**	**Mean Difference**	**SE**	**df**	**t**	p_{tukey}
**H. decurrens**	**March**	-	**H. decurrens**	**September**	16.70	4.43	68.0	3.766	0.002
		-	**A. dealbata**	**March**	-55.12	4.61	68.0	-11.946	<.001
		-	**A. dealbata**	**September**	18.24	4.61	68.0	3.953	0.001
	**September**	-	**A. dealbata**	**March**	-71.82	4.61	68.0	-15.564	<.001
		-	**A. dealbata**	**September**	1.55	4.61	68.0	0.335	0.987
**A. dealbata**	**March**	-	**A. dealbata**	**September**	73.36	4.79	68.0	15.321	<.001

Table S7: Results of Tukey's HSD test identifying differences in the allelopathic effects of invasive species on seed germination rate depending on the native species

Post Hoc Comparisons - Invasive species ✻ Native species

Comparison									
**Invasive species**	**Native species**		**Invasive species**	**Native species**	**Mean Difference**	**SE**	**df**	**t**	p_{tukey}
**H. decurrens**	**C. ladanifer**	**-**	**H. decurrens**	**L. stoechas**	27.52	4.43	68.0	6.21	<.001
		**-**	**A. dealbata**	**C. ladanifer**	-44.65	4.43	68.0	-10.07	<.001
		**-**	**A. dealbata**	**L. stoechas**	18.60	4.79	68.0	3.88	0.001
	**L. stoechas**	**-**	**A. dealbata**	**C. ladanifer**	-72.17	4.43	68.0	-16.28	<.001
		**-**	**A. dealbata**	**L. stoechas**	-8.93	4.79	68.0	-1.86	0.253
**A. dealbata**	**C. ladanifer**	**-**	**A. dealbata**	**L. stoechas**	63.25	4.79	68.0	13.21	<.001

Table S8: Results of Tukey's HSD test identifying significant differences in the germination ratio of native species depending on the month of origin of the plant material

Post Hoc Comparisons - Native species ✻ Month

Comparison									
**Native species**	**Month**		**Native species**	**Month**	**Mean Difference**	**SE**	**df**	**t**	p_{tukey}
**C. ladanifer**	**March**	**-**	**C. ladanifer**	**September**	71.783	4.43	68.0	16.1924	<.001
		**-**	**L. stoechas**	**March**	72.137	4.61	68.0	15.6339	<.001
		**-**	**L. stoechas**	**September**	90.412	4.61	68.0	19.5946	<.001
	**September**	**-**	**L. stoechas**	**March**	0.354	4.61	68.0	0.0768	1.000
		**-**	**L. stoechas**	**September**	18.629	4.61	68.0	4.0374	<.001
**L. stoechas**	**March**	**-**	**L. stoechas**	**September**	18.275	4.79	68.0	3.8166	0.002

Table S9: Results of the ANOVA analysis examining the effects of invasive species, native species, concentration, month, and their combined interaction on the hypocotyl emergence of native species seeds

ANOVA - Hypocotyls

	Sum of Squares	df	Mean Square	F	p
**Invasive species**	152.0	1	152.0	0.6513	0.422
**Native species**	1454.8	1	1454.8	6.2323	0.015
**Concentration**	98430.9	2	49215.4	210.8395	<.001
**Month**	161.0	1	161.0	0.6898	0.409
**Invasive species ✻ Native species**	8202.3	1	8202.3	35.1390	<.001
**Invasive species ✻ Concentration**	13116.1	2	6558.0	28.0947	<.001
**Native species ✻ Concentration**	221.7	2	110.8	0.4749	0.624
**Invasive species ✻ Month**	4930.0	1	4930.0	21.1200	<.001
**Native species ✻ Month**	22.8	1	22.8	0.0977	0.756
**Concentration ✻ Month**	153.1	2	76.6	0.3280	0.721
**Invasive species ✻ Native species ✻ Concentration**	1539.7	2	769.8	3.2980	0.043
**Invasive species ✻ Native species ✻ Month**	129.8	1	129.8	0.5561	0.458
**Invasive species ✻ Concentration ✻ Month**	1194.3	2	597.2	2.5583	0.084
**Native species ✻ Concentration ✻ Month**	1379.1	2	689.6	2.9541	0.058
**Invasive species ✻ Native species ✻ Concentration ✻ Month**	1316.4	2	658.2	2.8197	0.066
**Residuals**	16806.7	72	233.4		

Table S10: Results of Tukey's HSD test identifying significant differences in the allelopathic effects of invasive species on hypocotyl emergence depending on the month of origin of the plant material

Post Hoc Comparisons - Invasive species ✻ Month

Comparison									
**Invasive species**	**Month**		**Invasive species**	**Month**	**Mean Difference**	**SE**	**df**	**t**	p_{tukey}
**H. decurrens**	**March**	-	**H. decurrens**	**September**	16.9225	4.41	72.0	3.8369	0.001
		-	**A. dealbata**	**March**	16.8492	4.41	72.0	3.8203	0.002
		-	**A. dealbata**	**September**	5.1071	4.41	72.0	1.1579	0.655
	**September**	-	**A. dealbata**	**March**	-0.0733	4.41	72.0	-0.0166	1.000
		-	**A. dealbata**	**September**	-11.8154	4.41	72.0	-2.6790	0.044
**A. dealbata**	**March**	-	**A. dealbata**	**September**	-11.7421	4.41	72.0	-2.6623	0.046

Table S11: Results of Tukey's HSD test identifying differences in the allelopathic effects of invasive species on hypocotyl emergence depending on the native species

Post Hoc Comparisons - Invasive species ✻ Native species

Comparison									
**Invasive species**	**Native species**		**Invasive species**	**Native species**	**Mean Difference**	**SE**	**df**	**t**	p_{tukey}
**H. decurrens**	**C. ladanifer**	-	**H. decurrens**	**L. stoechas**	26.27	4.41	72.0	5.96	<.001
		-	**A. dealbata**	**C. ladanifer**	21.00	4.41	72.0	4.76	<.001
		-	**A. dealbata**	**L. stoechas**	10.30	4.41	72.0	2.34	0.099
	**L. stoechas**	-	**A. dealbata**	**C. ladanifer**	-5.27	4.41	72.0	-1.19	0.632
		-	**A. dealbata**	**L. stoechas**	-15.97	4.41	72.0	-3.62	0.003
**A. dealbata**	**C. ladanifer**	-	**A. dealbata**	**L. stoechas**	-10.70	4.41	72.0	-2.43	0.081

Table S12: Results of the ANOVA analysis examining the effects of invasive species, native species, concentration, month, and their combined interaction on the hypocotyl emergence rate of native species seeds

ANOVA - CV

	Sum of Squares	df	Mean Square	F	p
**Invasive species**	306979.7	1	306979.7	283.6137	<.001
**Native species**	15.6	1	15.6	0.0144	0.905
**Concentration**	508822.0	2	254411.0	235.0463	<.001
**Month**	24073.8	1	24073.8	22.2414	<.001
**Invasive species ✻ Native species**	1724.5	1	1724.5	1.5933	0.211
**Invasive species ✻ Concentration**	370137.7	2	185068.9	170.9822	<.001
**Native species ✻ Concentration**	19152.3	2	9576.2	8.8473	<.001
**Invasive species ✻ Month**	14337.4	1	14337.4	13.2461	<.001
**Native species ✻ Month**	17350.5	1	17350.5	16.0299	<.001
**Concentration ✻ Month**	13109.9	2	6554.9	6.0560	0.004
**Invasive species ✻ Native species ✻ Concentration**	37751.4	2	18875.7	17.4390	<.001
**Invasive species ✻ Native species ✻ Month**	15854.9	1	15854.9	14.6481	<.001
**Invasive species ✻ Concentration ✻ Month**	4064.1	2	2032.1	1.8774	0.161
**Native species ✻ Concentration ✻ Month**	13264.2	2	6632.1	6.1273	0.004
**Invasive species ✻ Native species ✻ Concentration ✻ Month**	5563.3	2	2781.7	2.5699	0.084
**Residuals**	73602.3	68	1082.4		

Table S13: Results of Tukey's HSD test assessing differences in the allelopathic effects of invasive species on the hypocotyl emergence rate of native species

Post Hoc Comparisons - Invasive species

Comparison							
**Invasive species**		**Invasive species**	**Mean Difference**	**SE**	**df**	**t**	p_{tukey}
**H. decurrens**	-	**A. dealbata**	-118	6.99	68.0	-16.8	<.001

Table S14: Results of Tukey's HSD test identifying significant differences in the allelopathic effects of invasive species on hypocotyl emergence rate depending on the month of origin of the plant material

Post Hoc Comparisons - Invasive species ✻ Month

Comparison									
**Invasive species**	**Month**		**Invasive species**	**Month**	**Mean Difference**	**SE**	**df**	**t**	p_{tukey}
**H. decurrens**	**March**	-	**H. decurrens**	**September**	7.53	9.50	68.0	0.792	0.858
		-	**A. dealbata**	**March**	-143.15	9.89	68.0	-14.482	<.001
		-	**A. dealbata**	**September**	-84.75	9.89	68.0	-8.573	<.001
	**September**	-	**A. dealbata**	**March**	-150.68	9.89	68.0	-15.243	<.001
		-	**A. dealbata**	**September**	-92.27	9.89	68.0	-9.335	<.001
**A. dealbata**	**March**	-	**A. dealbata**	**September**	58.40	10.26	68.0	5.693	<.001

Table S15: Results of Tukey's HSD test identifying significant differences in the hypocotyl emergence rate of native species depending on the month of origin of the plant material

Post Hoc Comparisons - Native species ✻ Month

Comparison									
**Native species**	**Month**		**Native species**	**Month**	**Mean Difference**	**SE**	**df**	**t**	**p_{tukey}**
**C. ladanifer**	**March**	**-**	**C. ladanifer**	**September**	60.95	9.50	68.0	6.418	<.001
		**-**	**L. stoechas**	**March**	27.15	9.89	68.0	2.746	0.038
		**-**	**L. stoechas**	**September**	32.13	9.89	68.0	3.250	0.010
	**September**	**-**	**L. stoechas**	**March**	-33.80	9.89	68.0	-3.420	0.006
		**-**	**L. stoechas**	**September**	-28.83	9.89	68.0	-2.916	0.024
**L. stoechas**	**March**	**-**	**L. stoechas**	**September**	4.98	10.26	68.0	0.485	0.962

Table S16: Results of the ANOVA analysis examining the effects of invasive species, native species, concentration, month, and their combined interaction on the root length of native species seeds

ANOVA - Root length

	Sum of Squares	df	Mean Square	F	p
**Invasive species**	152.5	1	152.5	0.4683	0.494
**Native species**	68.5	1	68.5	0.2103	0.647
**Concentration**	405408.4	2	202704.2	622.5357	<.001
**Month**	459.5	1	459.5	1.4113	0.235
**Invasive species ✻ Native species**	8989.1	1	8989.1	27.6068	<.001
**Invasive species ✻ Concentration**	23180.9	2	11590.4	35.5960	<.001
**Native species ✻ Concentration**	3120.6	2	1560.3	4.7919	0.009
**Invasive species ✻ Month**	1496.5	1	1496.5	4.5960	0.032
**Native species ✻ Month**	191.2	1	191.2	0.5871	0.444
**Concentration ✻ Month**	2446.0	2	1223.0	3.7560	0.024
**Invasive species ✻ Native species ✻ Concentration**	419.9	2	209.9	0.6448	0.525
**Invasive species ✻ Native species ✻ Month**	27.1	1	27.1	0.0831	0.773
**Invasive species ✻ Concentration ✻ Month**	2596.7	2	1298.3	3.9874	0.019
**Native species ✻ Concentration ✻ Month**	6653.0	2	3326.5	10.2162	<.001
**Invasive species ✻ Native species ✻ Concentration ✻ Month**	1803.7	2	901.8	2.7697	0.063
**Residuals**	304771.5	936	325.6		

Table S17: Results of Tukey's HSD test identifying differences in the allelopathic effects of invasive species on root length depending on the native species

Post Hoc Comparisons - Invasive species ✻ Native species

Comparison									
**Invasive species**	**Native species**		**Invasive species**	**Native species**	**Mean Difference**	**SE**	**df**	**t**	p_{tukey}
**H. decurrens**	**C. ladanifer**	-	**H. decurrens**	**L. stoechas**	6.654	1.65	936	4.040	<.001
		-	**A. dealbata**	**C. ladanifer**	5.323	1.65	936	3.231	0.007
		-	**A. dealbata**	**L. stoechas**	-0.263	1.65	936	-0.160	0.999
	**L. stoechas**	-	**A. dealbata**	**C. ladanifer**	-1.331	1.65	936	-0.808	0.851
		-	**A. dealbata**	**L. stoechas**	-6.917	1.65	936	-4.199	<.001
**A. dealbata**	**C. ladanifer**	-	**A. dealbata**	**L. stoechas**	-5.586	1.65	936	-3.391	0.004

Table S18: Results of Tukey's HSD test identifying differences in the allelopathic effects of invasive species on root length as a function of native species and the month of origin of the plant material

Post Hoc Comparisons - Invasive species ✻ Native species ✻ Month

Comparison											
**Invasive species**	**Native species**	**Month**		**Invasive species**	**Native species**	**Month**	**Mean Difference**	**SE**	**df**	**t**	**p_{tukey}**
**H. decurrens**	**C. ladanifer**	**March**	**-**	**H. decurrens**	**C. ladanifer**	**September**	0.557	2.33	936	0.239	1.000
			**-**	**H. decurrens**	**L. stoechas**	**March**	6.098	2.33	936	2.617	0.151
			**-**	**H. decurrens**	**L. stoechas**	**September**	7.768	2.33	936	3.334	0.020
			**-**	**A. dealbata**	**C. ladanifer**	**March**	8.156	2.33	936	3.501	0.011
			**-**	**A. dealbata**	**C. ladanifer**	**September**	3.047	2.33	936	1.308	0.896
			**-**	**A. dealbata**	**L. stoechas**	**March**	1.342	2.33	936	0.576	0.999
			**-**	**A. dealbata**	**L. stoechas**	**September**	-1.311	2.33	936	-0.563	0.999
		**September**	**-**	**H. decurrens**	**L. stoechas**	**March**	5.541	2.33	936	2.378	0.253
			**-**	**H. decurrens**	**L. stoechas**	**September**	7.211	2.33	936	3.095	0.042
			**-**	**A. dealbata**	**C. ladanifer**	**March**	7.599	2.33	936	3.262	0.025
			**-**	**A. dealbata**	**C. ladanifer**	**September**	2.490	2.33	936	1.069	0.963
			**-**	**A. dealbata**	**L. stoechas**	**March**	0.785	2.33	936	0.337	1.000
			**-**	**A. dealbata**	**L. stoechas**	**September**	-1.867	2.33	936	-0.802	0.993
	**L. stoechas**	**March**	**-**	**H. decurrens**	**L. stoechas**	**September**	1.670	2.33	936	0.717	0.997
			**-**	**A. dealbata**	**C. ladanifer**	**March**	2.058	2.33	936	0.884	0.988
			**-**	**A. dealbata**	**C. ladanifer**	**September**	-3.051	2.33	936	-1.310	0.895
			**-**	**A. dealbata**	**L. stoechas**	**March**	-4.756	2.33	936	-2.042	0.454
			**-**	**A. dealbata**	**L. stoechas**	**September**	-7.408	2.33	936	-3.180	0.033
		**September**	**-**	**A. dealbata**	**C. ladanifer**	**March**	0.388	2.33	936	0.167	1.000
			**-**	**A. dealbata**	**C. ladanifer**	**September**	-4.721	2.33	936	-2.026	0.464
			**-**	**A. dealbata**	**L. stoechas**	**March**	-6.426	2.33	936	-2.758	0.107
			**-**	**A. dealbata**	**L. stoechas**	**September**	-9.078	2.33	936	-3.897	0.003
**A. dealbata**	**C. ladanifer**	**March**	**-**	**A. dealbata**	**C. ladanifer**	**September**	-5.109	2.33	936	-2.193	0.357
			**-**	**A. dealbata**	**L. stoechas**	**March**	-6.814	2.33	936	-2.925	0.069
			**-**	**A. dealbata**	**L. stoechas**	**September**	-9.467	2.33	936	-4.064	0.001
		**September**	**-**	**A. dealbata**	**L. stoechas**	**March**	-1.705	2.33	936	-0.732	0.996
			**-**	**A. dealbata**	**L. stoechas**	**September**	-4.357	2.33	936	-1.871	0.572
	**L. stoechas**	**March**	**-**	**A. dealbata**	**L. stoechas**	**September**	-2.653	2.33	936	-1.139	0.948
